# Single-cell multi-omics dissection of c-Myb/AURKA-mediated autophagy and metabolic reprogramming in diabetic adipose-derived stem cells

**DOI:** 10.3389/fimmu.2025.1665909

**Published:** 2025-09-25

**Authors:** Yating Yin, Wenwen Shao, Wen Zha, Bin Wang

**Affiliations:** ^1^ Department of Plastic and Reconstructive Surgery, Shanghai Ninth People’s Hospital, Shanghai Jiao Tong University School of Medicine, Shanghai, China; ^2^ Department of Burn and Plastic Surgery, Seventh People’s Hospital Affiliated to Shanghai University of Traditional Chinese Medicine, Shanghai, China; ^3^ Shandong Traditional Chinese Medicine University, Shandong, China

**Keywords:** multi-omics integration, diabetes mellitus, adipose-derived stem cells, metabolic reprogramming, inflammatory disease

## Abstract

**Background:**

Diabetes mellitus (DM) alters the functional properties of adipose-derived stem cells (ADSCs), contributing to impaired tissue repair in diabetic foot ulcers (DFUs), a condition characterized by chronic inflammation. Although multi-omics studies have identified metabolic dysregulation in DM, the transcriptional and metabolic networks underlying ADSCs dysfunction remain elusive. Here, we integrated single-cell transcriptomics and metabolic profiling to characterize DM-associated ADSCs subpopulations and explored the effects of high glucose (HG)-induced inflammatory stress on autophagy, apoptosis, and metabolic reprogramming.

**Methods:**

We analyzed single-cell RNA sequencing (scRNA-seq) data from ADSCs of three DM patients and three healthy donors. Subpopulations were clustered using Seurat, and functional annotations were performed via enrichment analysis. Autophagy, apoptosis, and metabolic pathways were assessed using AUCell scoring. Experimental validation was conducted using HG-treated ADSCs, including c-Myb/AURKA overexpression/knockdown, Co-IP, ChIP, and dual-luciferase reporter assays.

**Results:**

We identified fourteen ADSCs subpopulations, among which C5 (TOP2A High), C8 (AURKA High), C9 (CCNB1 High), and C11 (MMP3 High) exhibited G2/M phase preference and enhanced stemness (C11) or proliferation (C8) in DM. HG induced autophagy in ADSCs via c-Myb/AURKA pathway to resist apoptosis. Mechanistically, c-Myb directly bound to the AURKA promoter, and AURKA knockdown abolished c-Myb-induced autophagy. Metabolic reprogramming shifted toward glycolysis/gluconeogenesis in DM, particularly in C8 subpopulation.

**Conclusions:**

Our study integrates multi-omics to demonstrate that DM induces distinct ADSCs subpopulations with dysregulated cell cycle, stemness, autophagy, apoptosis and metabolic profiles. HG activates c-Myb/AURKA-mediated autophagy in ADSCs, suggesting a potential regulatory mechanism in diabetic inflammatory microenvironments. Upregulating c-Myb may restore ADSCs function in DFUs, providing a foundation for future personalized therapies.

## Graphical Abstract

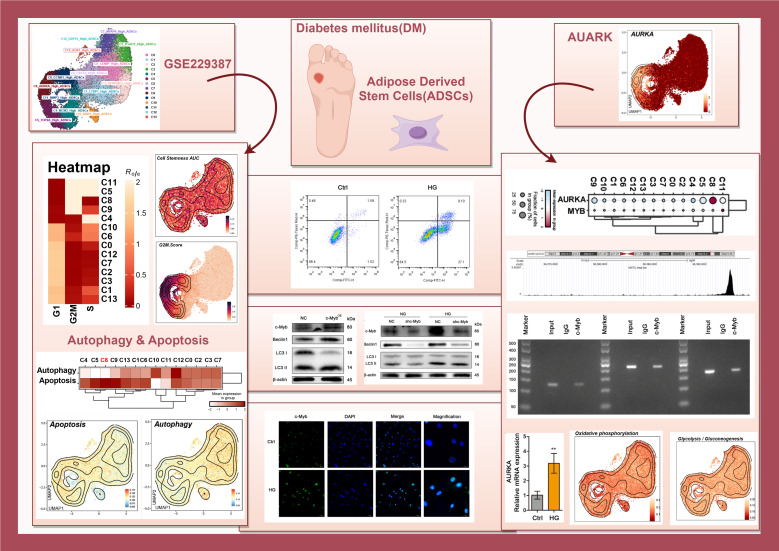

## 1 Introduction

Adipose Derived Stem Cells (ADSCs) based therapy represents a promising strategy for various regenerative medicine applications. Impaired diabetic wound healing, a major complication of diabetes, often progresses to chronic inflammatory ulcers and may culminate in diabetic foot syndrome, posing a significant threat to limb viability (1). Accumulating evidence demonstrates that ADSCs ameliorate diabetes mellitus (DM) by modulating insulin resistance and hyperglycemia, regulating glucose metabolism, promoting β-cell function and regeneration, and serving as gene delivery vectors (2). Moreover, ADSCs accelerate diabetic wound healing through collagen deposition, inflammation suppression, and angiogenesis promotion (3). However, their clinical translation remains limited.

High-throughput multi-omics techniques, including transcriptomics, proteomics, and metabolomics, enable the discovery of novel disease mechanisms and therapeutic targets. Data integration across these platforms is critical for deciphering complex biological systems and molecular networks (4). Although DM disrupts stem cell function via hyperglycemia-induced stress (5), the heterogeneous responses of ADSCs subpopulations to diabetic microenvironments are incompletely understood. Thus, we combined single-cell transcriptomics with metabolic profiling to elucidate key targets for diabetic ulcer therapy.

The transcription factor c-Myb regulates gene expression by binding to promoter regions (6). In tumors, c-Myb amplification induces GRP78 expression, promoting cell survival under hypoxia and nutrient deprivation (7). c-Myb plays a critical role in regulating hematopoietic stem/progenitor cell mobilization during zebrafish hematopoiesis (8). It is also involved in regulating the differentiation program of myogenic progenitor cells (9). Furthermore, c-Myb contributes to osteogenesis in long bones through the induction of osteogenic genes and promotion of mineralized matrix production (10). Similarly, Aurora kinase A (AURKA), a serine/threonine kinase essential for mitosis, attenuates diabetes-impaired angiogenesis and oxidative stress in ischemic tissues (11). Studies further indicate that AURKA from human Wharton’s jelly-derived MSCs (WJ-MSCs) regulated the antiapoptotic effect of skeletal muscle cells, suggesting its therapeutic potential for muscle diseases, such as Duchenne muscular dystrophy (12). Moreover, the low-dose inhibition of Aurora A extended the length of the primary cilium, restored the invasion and migration potential, and improved the differentiation capacity of obese ADSCs, highlighting a promising strategy for countering obesity-related metabolic diseases (13). Yet, their roles in ADSCs dysfunction under high glucose (HG) remain unexplored.

Here, we employed scRNA-seq to analyze ADSCs from DM patients and healthy controls, revealing 14 distinct subpopulations with distinct cell-cycle, metabolic signatures and stemness—the inherent capacity of ADSCs to self-renew and differentiate into multiple cell lineages (14). We further established that HG induces autophagy via the c-Myb/AURKA axis, while c-Myb overexpression confers apoptosis resistance. Additionally, HG shifts metabolism toward glycolysis. Our findings uncover subpopulation-specific dysregulation in diabetic ADSCs and implicate c-Myb/AURKA as a regulatory hub linking glucose stress to ADSCs dysfunction.

## 2 Results

### 2.1 scRNA-seq profiling of DM ADSCs

**Figure 1:**
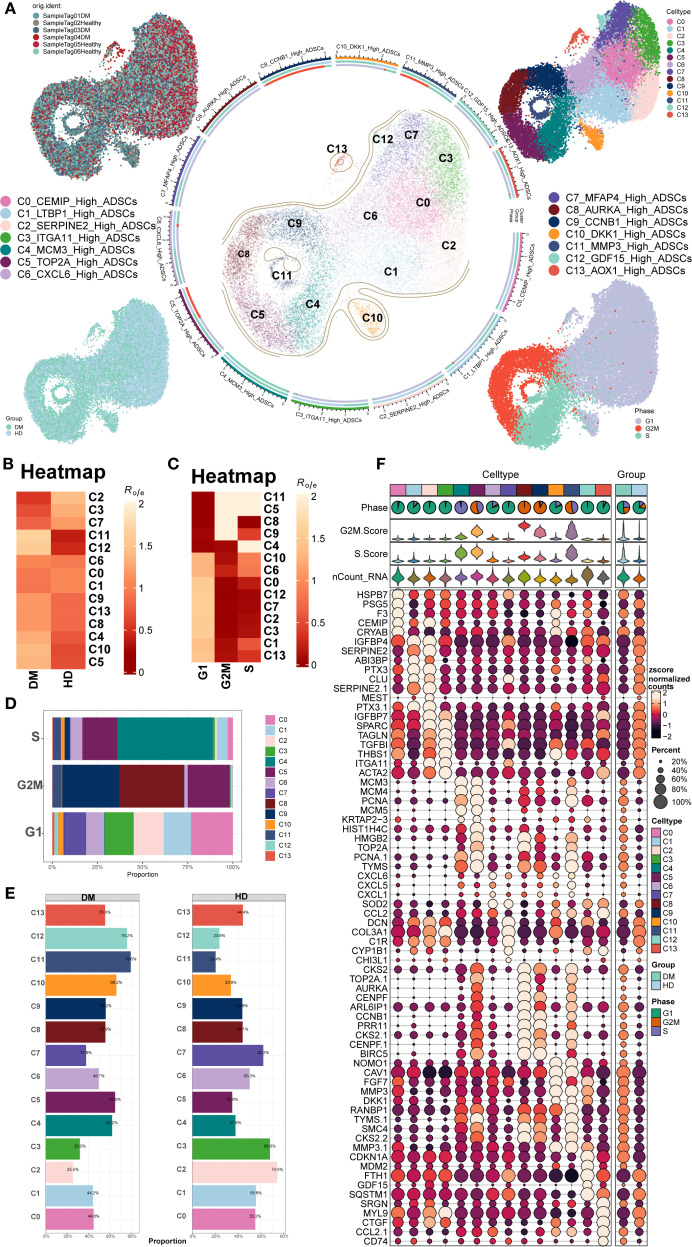
Overview of ADSCs subpopulations in DM. **(A)** The central circular plot illustrated the distribution of 14 DM ADSCs subpopulations. UMAP plots showed the sample origin, group classification (HD, healthy donor; DM, diabetes mellitus), and cell cycle phase of each cell. **(B, C)** The heatmaps depicted the tissue preference and cell cycle bias of the 14 DM ADSCs subpopulations. **(D)** The bar plot demonstrated the cell cycle distribution across the 14 DM ADSCs subpopulations. **(E)** The bar plot displayed the relative proportions of each ADSCs subpopulation across different groups. **(F)** Bubble plots presented the expression of the top five genes in each ADSCs subpopulation across different groups.

ScRNA-seq data (GSE229387) from ADSCs were analyzed, comprising samples from three DM patients (Tag01, Tag03, and Tag04) and three healthy donors (Tag02, Tag05, and Tag06). After quality control and removal of doublets, high-quality cells were retained and clustered into 14 Seurat clusters: C0 *CEMIP* High ADSCs, C1 *LTBP1* High ADSCs, C2 *SERPINE2* High ADSCs, C3 *ITGA11* High ADSCs, C4 *MCMS* High ADSCs, C5 *TOP2A* High ADSCs, C6 *CXCL6* High ADSCs, C7 *MFAP4* High ADSCs, C8 *AURKA* High ADSCs, C9 *CCNB1* High ADSCs, C10 *DKK1* High ADSCs, C11 *MMP3* High ADSCs, C12 *GDF15* High ADSCs, and C13 *AOX1* High ADSCs (**Figure 1A**).

UMAP plots were generated for different subpopulations across the healthy donor (HD) and DM groups and various cell cycle stages. The results showed that the C5 *TOP2A* High ADSCs, C8 *AURKA* High ADSCs, C9 *CCNB1* High ADSCs, and C11 *MMP3* High ADSCs subpopulations predominantly occupied the G2M phase. Additionally, the distribution of each DM ADSCs subpopulation across groups and cell cycle preferences was quantified by calculating the Ro/e ratio. This analysis confirmed that the C5, C8, C9, and C11 DM ADSCS subpopulations preferentially resided in the G2M phase (**Figures 1B, C**).

To further analyze the proportion of different subpopulations across cell cycle and group, proportion bar plots were generated (**Figures 1D, E**). The results revealed that, among all ADSCs subpopulations in the G2M phase, the C8 *AURKA* High ADSCs subpopulation had the highest proportion, followed by C9 *CCNB1* High ADSCs, with C5 *TOP2A* High ADSCs. The distribution of subpopulations across the groups also varied, with the C12 *GDF15* High ADSCs subpopulation comprising 76.2% in the DM group and 23.8% in the HD group, while the C11 *MMP3* High ADSCs subpopulation accounted for 79.6% in the DM group and 20.4% in the HD group.

The top five gene expressions for different subpopulations were shown in **Figure 1F**.

### 2.2 Stemness features and differential expression analysis of DM ADSCs subpopulations

**Figure 2:**
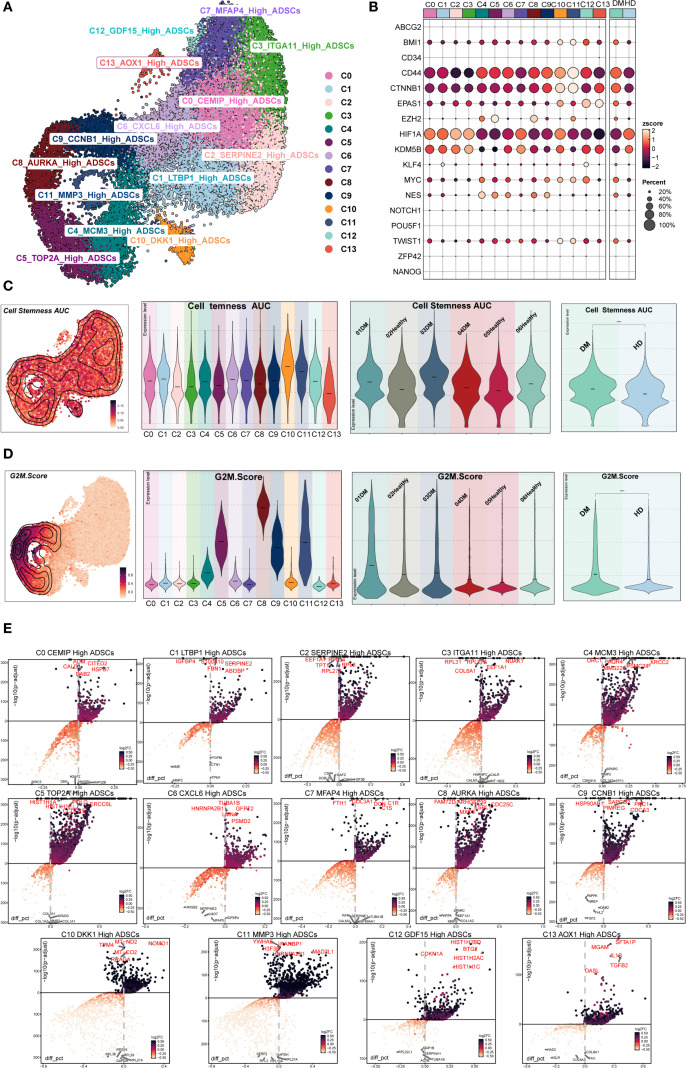
Stemness and differential gene expression analysis of DM ADSCs subpopulations. **(A)** The UMAP plot illustrated the distribution of 14 ADSCs subpopulations. **(B)** The bubble plot showed the expression of stemness-related genes across ADSCs subpopulations and groups. **(C)** The UMAP plot depicted the distribution of Cell Stemness AUC scores; violin plots presented the stemness levels across subpopulations, individual samples, and groups. **(D)** The UMAP plot showed the distribution of G2M scores; violin plots illustrated G2M scores across subpopulations, individual samples, and groups. **(E)** The volcano plots identified upregulated and downregulated genes across the 14 DM ADSCs subpopulations.

**Figure 2A** illustrated the distribution of DM ADSCs subpopulations. Analysis of stemness-associated gene expression profiles (**Figure 2B**) revealed that CD44, CTNNB1, HIF1A, and KDM5B were highly expressed across multiple DM ADSCs subpopulations, suggesting their broad involvement in the maintenance of ADSCs stemness.

To further evaluate stemness characteristics, we calculated the Cell Stemness AUC and G2M Score across different DM ADSCs subpopulations, individual patients, and groups (**Figures 2C, D**). The C11 *MMP3* High ADSCs subpopulation exhibited the highest Cell Stemness AUC, indicating strong stemness potential. At the individual level, Sample Tag03DM showed the highest stemness score among all samples, and the DM group demonstrated elevated stemness compared to the HD group.

Regarding cell cycle activity, the C8 *AURKA* High ADSCs subpopulation had the highest G2M Score, with Sample Tag01DM showing the most pronounced G2M activity among all samples. Overall, the DM group displayed increased G2M scores compared to the HD group, suggesting that the diabetic state might enhance the proliferative activity of specific ADSCs subpopulations.

Differential expression analysis further identified characteristic upregulated genes in the C8 *AURKA* High ADSCs subpopulation, which exhibited the highest G2M Score. These included *FAM72D, ARHGEF39, ESPL1, MXD3*, and *CDC25C* (**Figure 2E**). These genes are broadly involved in cell cycle regulation and stemness maintenance, potentially conferring distinct functional properties to this subpopulation within the diabetic microenvironment.

### 2.3 Biological functions of DM ADSCs subpopulations

**Figure 3:**
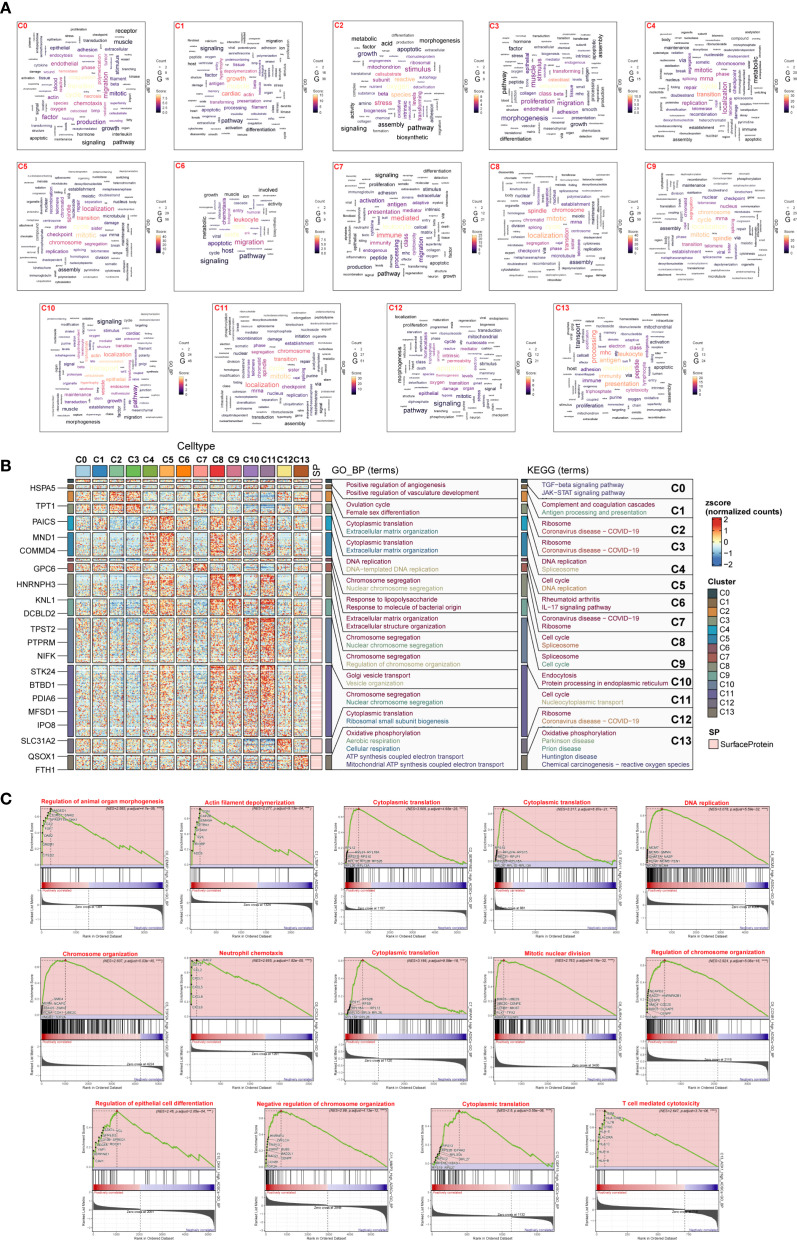
Enrichment analysis of DM ADSCs subpopulations. **(A)** Word cloud diagrams illustrated enriched terms in the 14 DM ADSCs subpopulations based on gene counts. **(B)** GO biological process (GOBP) and KEGG pathway enrichment analyses were performed for the 14 DM ADSCs subpopulations. **(C)** GSEA revealed enriched pathways in the 14 subpopulations, with only the top terms based on the highest normalized enrichment scores (NES) shown. (***p < 0.001, ****p < 0.0001).

To investigate the biological functions of different subpopulations of ADSCs in DM patients, we performed enrichment analysis on 14 ADSCs subpopulations (**Figures 3A–C**; **Supplementary Figure S1**). The results revealed distinct biological features and signaling pathways for each subpopulation.

C0 *CEMIP* High ADSCs were associated with coagulation and angiogenesis, involving TGF-β and JAK-STAT pathways, and played a role in organ morphogenesis. C1 *LTBP1* High ADSCs were linked to muscle and growth, participating in the ovulation cycle and complement cascades, and were associated with actin filament depolymerization. C2 *SERPINE2* High ADSCs were related to oxygen and reactive processes, involved in cytoplasmic translation and extracellular matrix organization, and implicated in ribosome function. C3 *ITGA11* High ADSCs were associated with osteoblasts, extracellular matrix organization, and ribosome functions. C4 *MCMS* High ADSCs were linked to the cell cycle, participating in DNA replication, and involved in the spliceosome. C5 *TOP2A* High ADSCs were related to mitosis, involved in chromosome segregation, and regulated chromosome organization. C6 *CXCL6* High ADSCs were associated with leukocytes, responding to lipopolysaccharide, and linked to rheumatoid arthritis and IL-17 signaling pathways. C7 *MFAP4* High ADSCs were associated with leukocytes, extracellular matrix and structure organization, and cytoplasmic translation. C8 *AURKA* High ADSCs were linked to chromosomes, involved in chromosome segregation, and regulated mitotic nuclear division. C9 *CCNB1* High ADSCs were related to mitosis, involved in chromosome segregation, and regulated chromosome organization. C10 *DKK1* High ADSCs were associated with localization, participating in Golgi vesicle transport and endocytosis. C11 *MMP3* High ADSCs were linked to mitosis, involved in chromosome segregation, and regulated chromosome organization. C12 *GDF15* High ADSCs were associated with apoptosis, involved in cytoplasmic translation and ribosome function. C13 *AOX1* High ADSCs were related to antigen processes, involved in oxidative phosphorylation, and participated in T cell-mediated cytotoxicity.

These enrichment analyses revealed the potential functions of different subpopulations of diabetes-related ADSCs, providing important biological insights into the impact of diabetes on ADSCs.

### 2.4 High glucose differentially regulated autophagy and apoptosis in ADSCs

**Figure 4:**
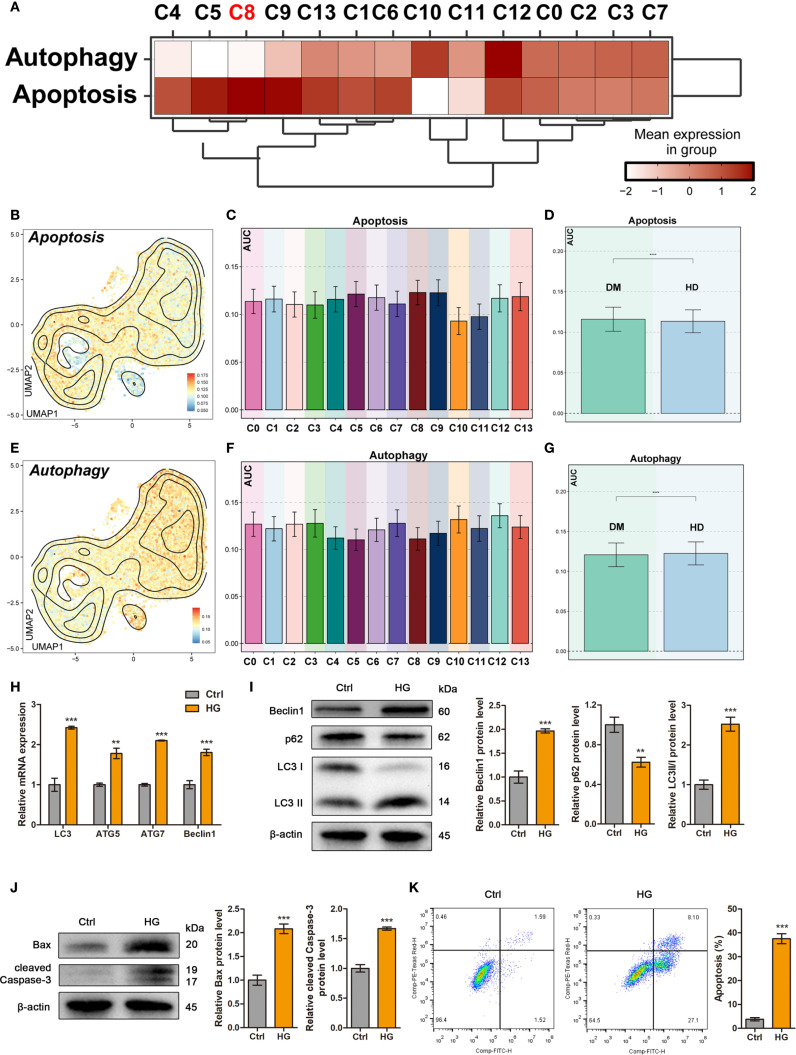
High glucose differentially regulated autophagy and apoptosis in ADSCs. **(A)** The heatmap showed the mean expression levels of Autophagy- and Apoptosis-related genes across DM ADSCs subpopulations. **(B-D)** UMAP plots and bar graphs illustrated the distribution and relative expression of Apoptosis-related gene sets across DM ADSCs subpopulations. **(E-G)** UMAP plots and bar graphs depicted the distribution and relative expression of Autophagy-related gene sets across DM ADSCs subpopulations. **(H)** RT–qPCR analysis of autophagy-related mRNAs in ADSCs treated with or without high glucose. **(I)** Western blot analysis of autophagy-related proteins in ADSCs treated with or without high glucose. β-actin was used as an internal control. **(J)** Western blot analysis of apoptosis-related proteins in ADSCs treated with or without high glucose. β-actin was used as an internal control. **(K)** Flow cytometry analysis and quantitative assessment of cell apoptosis in ADSCs treated with or without high glucose. Data are presented as mean ± SD from three independent biological replicates (n = 3). (**p < 0.01, ***p < 0.001, ****p < 0.0001).

To investigate the alterations of autophagy and apoptosis in ADSCs under diabetic conditions, we first scored the expression of autophagy- and apoptosis-related genes in each subpopulation based on scRNA-seq data. Compared to other subpopulations, the C5 *TOP2A* High ADSCs, C8 *AURKA* High ADSCs and C9 *CCNB1* High ADSCs exhibited elevated apoptosis scores and relatively low autophagy scores (**Figure 4A**).

To further investigate these differences, we applied the AUCell algorithm to quantify pathway activity scores in different subpopulations and between DM and HD groups. Apoptosis scores were significantly elevated in the C8 *AURKA* High ADSCs and C9 *CCNB1* High ADSCs subpopulations. Moreover, ADSCs from DM tissues displayed higher overall apoptosis scores than those from HD tissues, with statistical significance (**Figures 4B–D**).

In contrast, autophagy scores were generally similar in most DM ADSCs subpopulations (**Figures 4E, F**). Notably, HD-derived ADSCs showed higher overall autophagy scores than those from DM tissues (**Figure 4G**), suggesting a potential autophagy impairment in endogenous ADSCs under diabetic conditions, which may contribute to diabetic wound unhealing.

To investigate the effects of high glucose on exogenous ADSCs, cells were exposed to high glucose conditions to simulate the early diabetic microenvironment. Quantitative analysis demonstrated that high glucose treatment significantly upregulated autophagy-related genes, including *LC3, ATG5, ATG7*, and *BECLIN1* compared to the control group (**Figure 4H**). Consistent with transcriptional changes, western blot analysis confirmed that high glucose promoted autophagic activity, as evidenced by increased Beclin1 expression, enhanced LC3-I-to-LC3-II conversion, and degradation of p62 within 24 hours (**Figure 4I**). Furthermore, flow cytometry and western blotting revealed that high glucose triggered apoptosis in ADSCs (**Figures 4J, K**).

Taken together, these results indicate that high-glucose stress simultaneously induces autophagy and apoptosis in ADSCs, with subpopulation-specific susceptibility. This dual regulatory mechanism suggests that the diabetic microenvironment may compromise ADSCs function through coordinated activation of autophagic and apoptotic pathways.

### 2.5 High glucose enhanced autophagy in ADSCs via c-Myb upregulation

**Figure 5:**
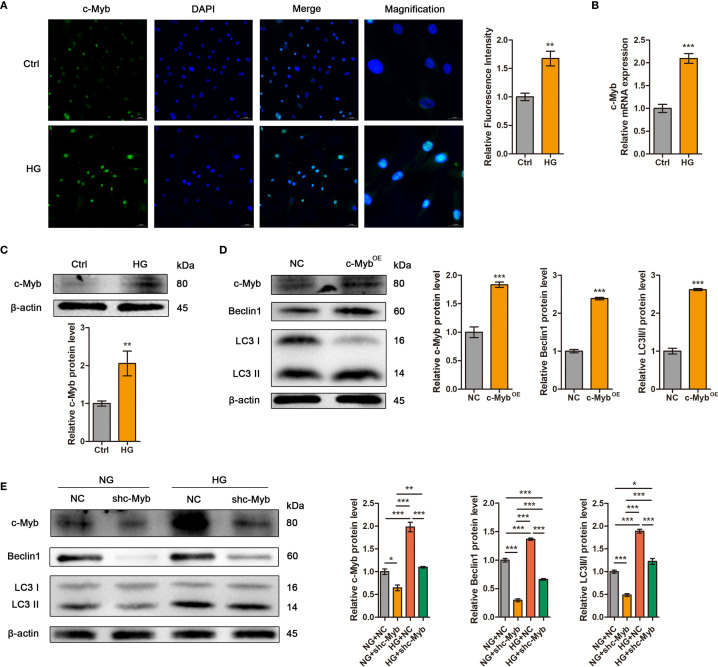
High glucose enhanced autophagy in ADSCs via c-Myb upregulation. **(A)** The expression of c-Myb was determined by immunofluorescence assay. Quantitative analysis was performed by measuring the mean fluorescence intensity of c-Myb. The nuclei were stained blue using DAPI. Scale bar = 50 mm. Magnification, Scale bar = 20 mm. **(B)** RT-qPCR analysis of c-Myb mRNA in ADSCs treated with or without high glucose. **(C)** Western blot analysis of c-Myb protein in ADSCs treated with or without high glucose. β-actin was used as an internal control. **(D)** Western blot analysis of c-Myb and autophagy-related proteins in ADSCs infected with c-Myb plasmid and vector control. β-actin was used as an internal control. OE, Overexpression. **(E)** Western blot analysis of c-Myb and autophagy-related proteins in ADSCs infected with c-Myb shRNA or vector control in normal glucose (NG) or high glucose (HG). β-actin was used as an internal control. Data are presented as mean ± SD from three independent biological replicates (n = 3). (*p < 0.05, **p < 0.01, ***p < 0.001).

Immunofluorescence analysis confirmed nuclear translocation of c-Myb in high glucose-treated ADSCs (**Figure 5A**). Consistent with this observation, both mRNA and protein levels of c-Myb were significantly elevated under high glucose conditions (**Figures 5B, C**). To investigate the effect of c-Myb on autophagy, we modulated c-Myb expression and assessed autophagic activity. c-Myb overexpression potentiated autophagy (**Figure 5D**), whereas c-Myb knockdown abrogated high glucose-induced upregulation of autophagy-related proteins in ADSCs (**Figure 5E**). These findings demonstrate that c-Myb is sufficient for high glucose-mediated autophagy activation in ADSCs.

### 2.6 c-Myb transcriptionally activates AURKA to mediate autophagy in ADSCs

**Figure 6:**
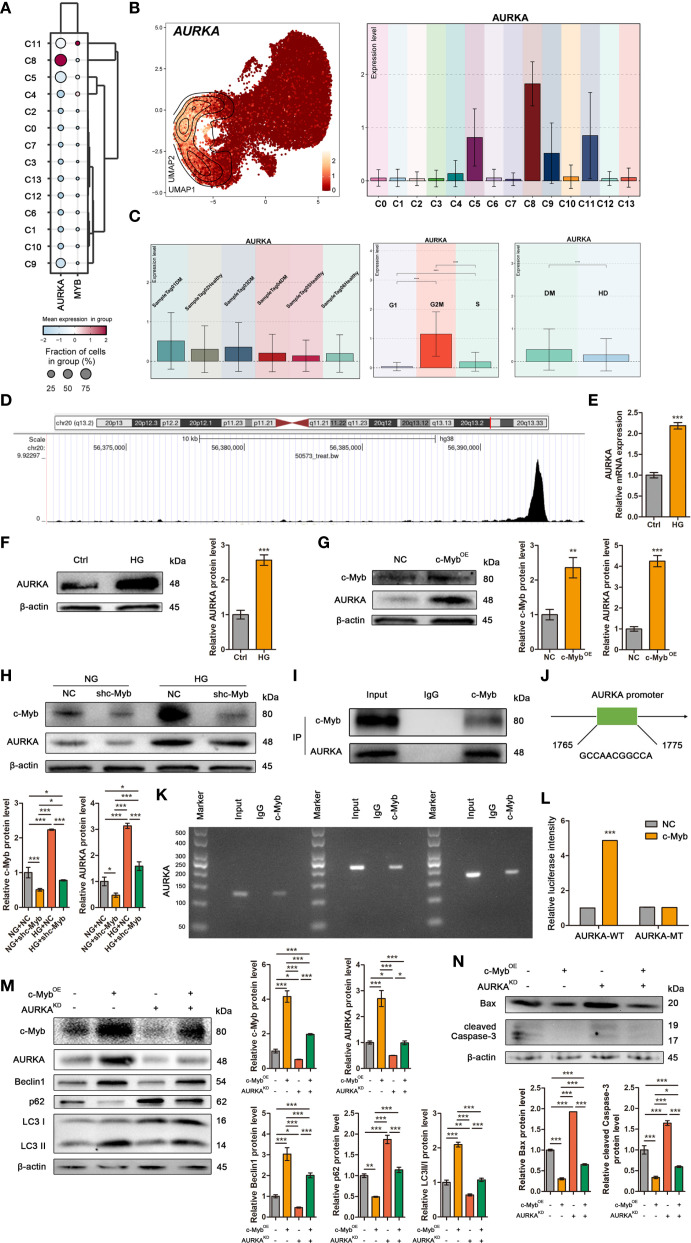
c-Myb transcriptionally activates AURKA to mediate autophagy in ADSCs. **(A)** The bubble plot showed the mean expression levels of AURKA and MYB across the 14 DM ADSCS subpopulations. **(B)** UMAP and bar plots revealed the distribution and expression levels of AURKA across 14 subpopulations. **(C)** Bar plots presented AURKA expression across different patient samples, cell cycle phases, and groups. **(D)** One genomic region in AURKA that exhibited increased c-Myb ChIP–seq signal in chromatin. **(E)** RT–qPCR analysis of AURKA mRNA in ADSCs treated with or without high glucose. **(F)** Western blot analysis of AURKA protein in ADSCs treated with or without high glucose. β-actin was used as an internal control. **(G)** Western blot analysis of c-Myb and AURKA proteins in ADSCs infected with c-Myb plasmid and vector control. β-actin was used as an internal control. OE, Overexpression. **(H)** Western blot analysis of c-Myb and AURKA proteins in ADSCs infected with c-Myb shRNA or vector control under normal glucose (NG) or high glucose (HG). β-actin was used as an internal control. **(I)** Co-IP assay to detect the interaction between c-Myb and AURKA. **(J)** The predicted binding site of c-Myb in the AURKA promoter region from the JASPAR database (relative profile score threshold 90%). **(K)** ChIP assay to analyze promoter co-occupancy by c-Myb and AURKA. **(L)** Luciferase reporter assay of c-Myb promoter activity with WT or mutated AURKA binding element (FHRE) in ADSCs. WT, Wild Type; MT, Mutant Type. **(M)** Western blot analysis of c-Myb, AURKA, and autophagy-related proteins in ADSCs infected with c-Myb plasmid, AURKA shRNA, or vector control. OE, Overexpression; KD, Knockdown. **(N)** Western blot analysis of apoptosis-related proteins in ADSCs infected with c-Myb plasmid, AURKA shRNA, or vector control. OE, Overexpression; KD, Knockdown. Data are presented as mean ± SD from three independent biological replicates (n = 3). (*p < 0.05, **p < 0.01, ***p < 0.001).

Transcriptomic analysis revealed broad AURKA expression across DM ADSCS subsets, with highest enrichment in the C8 subpopulation (**Figure 6A**). UMAP plot and bar plot analyses further confirmed elevated AURKA expression in C5, C8, C9, and C11 subsets (**Figure 6B**). Notably, DM-derived ADSCs exhibited significantly higher AURKA expression than HD-derived cells, particularly in samples Tag01 and Tag03, with peak expression during the G2/M phase (**Figure 6C**), suggesting AURKA’s role in high glucose stress responses in ADSCs.

To elucidate the molecular mechanisms underlying c-Myb-mediated regulation of autophagy in ADSCs under diabetic conditions, we initially performed target prediction analysis. ChIP-seq data mining (Cistrome Data Browser: 50573) identified AURKA as a potential direct transcriptional target of c-Myb (**Figure 6D**). Subsequent experimental validation demonstrated that high glucose treatment significantly upregulated both mRNA and protein expression levels of AURKA in ADSCs (**Figures 6E, F**). Further mechanistic investigations revealed that c-Myb overexpression markedly enhanced AURKA protein expression (**Figure 6G**). c-Myb knockdown effectively abolished high glucose-induced AURKA upregulation (**Figure 6H**). Co-IP assays revealed that c-Myb could directly interact with AURKA (**Figure 6I**). Bioinformatic analysis using JASPAR (http://jaspar.genereg.net/) predicted specific c-Myb binding sites within the AURKA promoter region (**Figure 6J**). This prediction was experimentally validated. ChIP-qPCR followed by agarose gel electrophoresis demonstrated direct binding of c-Myb to the AURKA promoter (**Figure 6K**). Furthermore, luciferase reporter assays comparing wild-type (AURKA-WT) and mutant (AURKA-MT with disrupted c-Myb binding site) promoter constructs revealed that the mutation completely abolished transcriptional regulation (**Figure 6L**). Together, these results provide conclusive evidence that c-Myb directly transcriptionally regulates AURKA expression. Functional characterization of the c-Myb/AURKA axis revealed its critical role in maintaining ADSCs homeostasis under diabetic conditions. Genetic ablation of AURKA not only attenuated c-Myb-mediated induction of autophagy-related proteins but also significantly increased apoptotic cell death in ADSCs (**Figures 6M, N**). These findings establish a mechanistic link whereby c-Myb, through transcriptional activation of AURKA, orchestrates a coordinated cellular response that promotes autophagic flux and enhances cell survival under high-glucose stress.

### 2.7 ADSCs exhibited subtype-specific metabolic reprogramming under diabetic conditions

**Figure 7:**
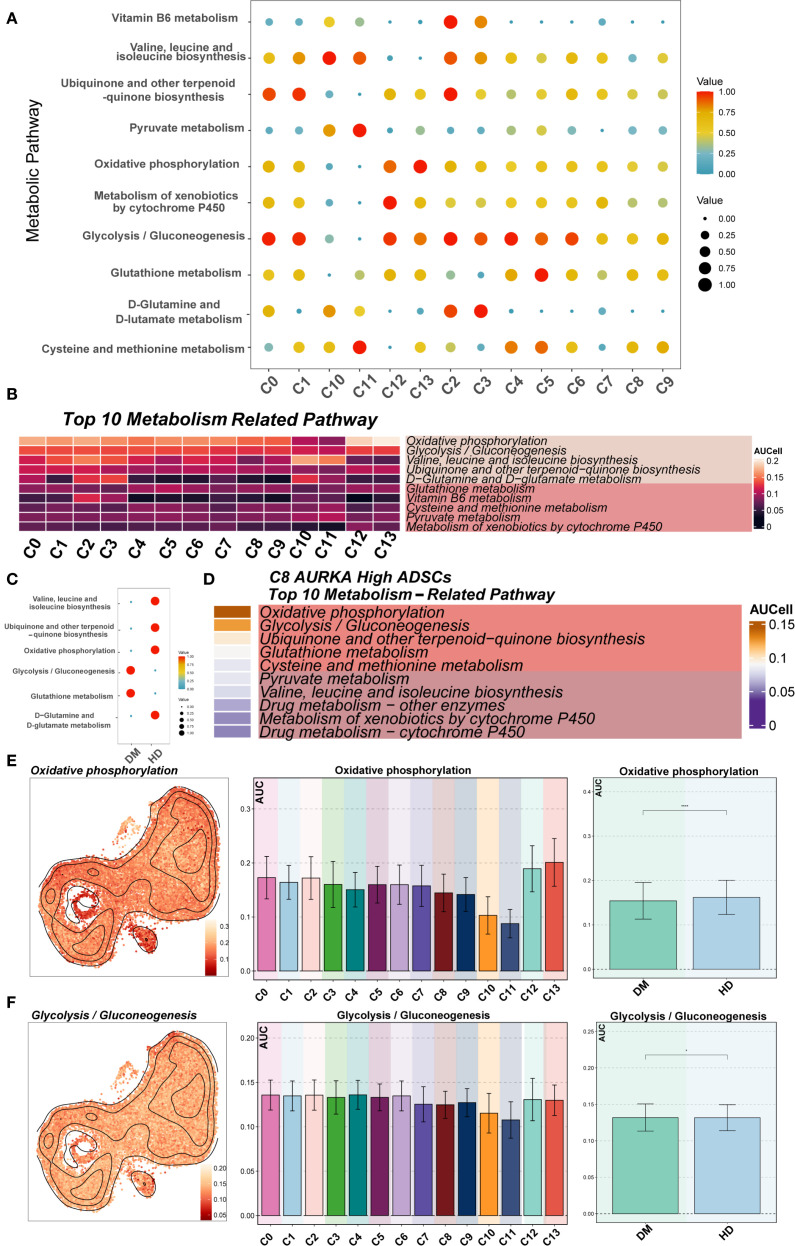
Metabolic pathway analysis of DM ADSCs subpopulations. **(A, B)** The bubble plot and heatmap showed the top 10 enriched metabolism-related pathway scores across the 14 DM ADSCs subpopulations. **(C)** The bubble plot illustrated the scores of metabolism-related pathways across different groups. **(D)** The heatmap displayed the top 10 enriched metabolic pathways in the C8 AURKA ADSCs subpopulation. **(E)** UMAP plots and bar graphs depicted the distribution and differential activity of the Oxidative phosphorylation pathway across DM ADSCs subpopulations and groups. **(F)** UMAP plots and bar graphs showed the distribution and differential activity of the Glycolysis/Gluconeogenesis pathway across DM ADSCs subpopulations and groups. (*p < 0.05, ***p < 0.001, ****p < 0.0001).

To systematically analyze the metabolic characteristics of ADSCs in the diabetic microenvironment, metabolic pathway scoring was performed on various DM ADSCS subpopulations and samples from different groups (**Figures 7A–C**). The results indicated that Oxidative phosphorylation and Glycolysis/Gluconeogenesis scored highly in most DM ADSCS subpopulations, suggesting they are the primary metabolic pathways in ADSCs. Further comparison revealed that Glycolysis/Gluconeogenesis was significantly elevated in the DM group, while Oxidative phosphorylation was more active in the HD group, indicating that diabetes may drive ADSCS metabolism toward glycolysis dependence.

In the key functional subpopulation, C8 *AURKA* High ADSCs, pathway analysis showed that the top two enriched pathways were Oxidative phosphorylation and Glycolysis/Gluconeogenesis (**Figure 7D**), suggesting significant metabolic activity in this subpopulation under the diabetic environment. Further analysis of the enrichment of Oxidative phosphorylation in different subpopulations revealed that C12 *GDF15* High ADSCs and C13 *AOX1* High ADSCs exhibited the highest enrichment in this pathway, while C10 *DKK1* High ADSCs and C11 *MMP3* High ADSCs had the lowest scores (**Figure 7E**). In contrast, Glycolysis/Gluconeogenesis showed more balanced distribution across the subpopulations (**Figure 7F**).

Overall, these results highlight a potential metabolic reprogramming trend in ADSCs within the diabetic microenvironment. The specific mechanisms and their impact on cell fate warrant further investigation.

## 3 Discussion

The diabetic wound microenvironment, characterized by severe inflammation, biofilm formation, excessive reactive oxygen species (ROS) production, hypoxia, and impaired nitric oxide (NO) synthesis, predisposes to chronic non-healing wounds (15). Comorbidities including peripheral vascular disease and polymicrobial infections further exacerbate this condition (16). Current clinical interventions such as debridement, growth factor therapy, and antimicrobial treatments often fail to achieve complete wound closure, underscoring the need for novel therapeutic targets (17).

ScRNA-seq has revolutionized our understanding of cellular heterogeneity, overcoming the limitations of bulk tissue analysis. When integrated with AI-driven multi-omics platforms, this technology enables precise decoding of disease-specific immune-inflammatory networks, paving the way for personalized medicine (18). Previous single-cell studies have revealed unprecedented details of diabetic foot ulcers (DFUs) pathophysiology (19, 20). Analyses of signaling pathways in DFUs have revealed that cytokines including IL-1, IL-16, LIGHT, CHEMERIN, and IGF are specifically expressed in non-healing wound tissues (21). scRNA-seq of healing enriched-fibroblasts in wound healing of DFU patients showed that fibroblasts overexpressing MMP1, MMP3, MMP11, HIF1A, CHI3L1 and TNFAIP6 genes exhibit pro-healing functions (22). Furthermore, stem cells serve as essential “seed cells” in wound repair by recruiting macrophages and endothelial lineage cells to ischemic and injured areas, where they facilitate tissue regeneration through the secretion of growth factors and establishment of a pro-regenerative microenvironment (23). Our study expands this knowledge by constructing a comprehensive single-cell atlas of diabetic ADSCs, identifying 14 functionally distinct subpopulations with altered cell-cycle distributions and metabolic preferences. Notably, the C5 *TOP2A* High ADSCs, C8 *AURKA* High ADSCs, C9 *CCNB1* High ADSCs, and C11 *MMP3* High ADSCs subpopulations showed predominant G2M phase accumulation under diabetic conditions, with C8 exhibiting the highest proportion. Cell cycle regulators including FAM72D, ARHGEF39, ESPL1, MXD3, and CDC25C may maintain stemness and confer unique functional adaptations to the diabetic microenvironment. Functional enrichment analysis revealed the potential functions of different subpopulations of diabetes-related ADSCs. Among these subpopulations, C8 *AURKA* High ADSCs were linked to chromosomes, involved in chromosome segregation, and regulated mitotic nuclear division. AURKA expression was significantly up-regulated in skin tissue samples from DFU patients compared to non-diabetic controls, suggesting its potential utility as a diagnostic or monitoring biomarker for DFU progression (24). Furthermore, AURKA overexpression rescued diabetes-related impairment of angiogenesis, arteriogenesis, and functional recovery in the ischemic limb (11). Conversely, pharmacological suppression of AURKA improved insulin sensitivity in a type 2 diabetic mouse model, an effect attributed in part to reduced macrophage infiltration and lower IL-6 levels (25).

When cellular damage exceeds repair capacity, programmed cell death pathways, including apoptosis, autophagy, pyroptosis and ferroptosis, are activated (26). Our data demonstrated elevated apoptosis in diabetic ADSCs, particularly in the C8 *AURKA* High ADSCs and C9 *CCNB1* High ADSCs subpopulations. The C8 subpopulation showed heightened susceptibility to high glucose-induced apoptosis, suggesting specific vulnerability to diabetic stress. While AURKA’s role in enhancing autophagy for wound repair is established (27), our findings extend its known mitotic functions (28) by revealing its critical role in ADSC survival under metabolic stress. Previous studies have demonstrated that autophagy plays a multifaceted role in all phases of wound healing, which exerts multiple protective effects, including preventing infection and excessive inflammation-induced tissue damage (29), inhibiting apoptosis and oxidative stress to enhance cell survival (5), and inducing keratinocyte differentiation, proliferation, and migration to promote wound re-epithelialization under HG environment (30). Diabetes mellitus significantly exacerbates neurological damage following spinal cord injury, both *in vivo* and *in vitro*, concomitant with enhanced apoptosis and increased autophagy activation (31). In contrast, diabetic foot ulcer tissues exhibit increased apoptosis alongside reduced autophagy (32), indicating tissue-specific differences in the autophagic response to diabetes. Our novel finding demonstrated that autophagy deficiency in endogenous ADSCs contributes to impaired diabetic wound healing.

The therapeutic potential of ADSCs and their exosomes for diabetic wounds is well-documented, with demonstrated efficacy in immunomodulation, cell recruitment, ECM remodeling, angiogenesis, and neuroregeneration (33, 34). As autophagy and apoptosis critically regulate stem cell survival and function (35, 36), various preconditioning strategies of ADSCs and ADSCs-derived exosomes prior to transplantation have been developed to enhance therapeutic outcomes, including specific gene overexpression (37), hypoxia pretreatment (38), low-glucose culture environment (39), and hydrogel encapsulation (40). In our study, the observed autophagy in diabetic ADSCs likely represents an initial protective response. Apoptotic cell death typically occurs when cellular stress exceeds the reparative capacity of autophagy. Consequently, numerous studies have focused on enhancing autophagic activity to promote diabetic wound healing (41, 42). The c-Myb transcription factor plays a role in skin development, and c-myb deficiency impaired wound repair and collagen type I levels (43). c-Myb could protect human dental pulp cells against glucose oxidative stress and regulate autophagy activity for pulp vitality via p-AMPK/AKT signaling (44). Moreover, c-Myb could regulate keratinocyte proliferation and migration under high glucose, influencing wound healing (45). Our study demonstrated c-Myb as a key regulator of ADSCs homeostasis through the c-Myb/AURKA axis in HG condition. Transplanted ADSCs initially activated autophagy as an adaptive survival response to diabetic stress. This transient autophagy boost may create a therapeutic window and overexpression of c-Myb may induce autophagy to resist apoptosis to secrete pro-healing factors before succumbing to diabetic stress. Our mechanistic studies confirmed direct c-Myb binding to the AURKA promoter, with AURKA knockdown abolishing c-Myb-mediated autophagy induction. This c-Myb/AURKA axis likely contributes to ADSCs survival in diabetic wound by disrupting the balance between autophagy and apoptosis. Targeting the c-Myb/AURKA axis, either through pharmacological or genetic means, may represent a promising future direction for developing novel therapeutic interventions to improve ADSCs-based therapies in diabetic wound healing.

Diabetes-associated metabolic dysregulation extends beyond hyperglycemia to involve complex network disturbances (46). Multiple metabolic pathways, including glycolysis, hexosamine biosynthesis, protein kinase C signaling, polyol metabolism, and advanced glycation end-product (AGE) formation, exhibit pro-oxidative properties and are frequently hyperactivated under diabetic conditions (47). Our metabolic profiling revealed preferential glycolysis over oxidative phosphorylation in DM ADSCs. Hyperglycemia and insulin resistance induce metabolic stress, causing mitochondrial dysfunction and disrupting energy homeostasis. Under these pathological conditions, HIF-1α becomes activated, orchestrating a metabolic shift by upregulating the expression of glycolytic enzymes, such as lactate dehydrogenase A, while concurrently suppressing oxidative phosphorylation pathways (48). Concurrently, enhanced activities of glycogen phosphorylase, fructose-1,6-bisphosphatase, and glucose-6-phosphatase promote glycogen breakdown, further increasing intracellular glucose availability (49, 50). The resultant glycolytic flux accelerates glucose utilization and leads to the accumulation of reactive metabolites such as methylglyoxal (MGO). Inefficient detoxification of MGO by glyoxalase 1 (GLO1) under diabetic conditions contributes to impaired tissue repair, including delayed wound healing (51, 52). Therefore, the glycolytic preference of DM ADSCs likely represents an adaptive response to the diabetic pathological microenvironment, where cells may sacrifice energy efficiency for rapid ATP generation to ensure survival under metabolic stress. However, excessive reliance on glycolysis could lead to functional impairments, such as a pro-inflammatory phenotype. The pronounced metabolic reprogramming observed in the C12 *GDF15* High ADSCs and C13 *AOX1* High ADSCs subpopulations suggests their potential role as key contributors to diabetic complications, making them promising therapeutic targets for further investigation.

We acknowledge that a key limitation of this study is the modest cohort size (n=3 per group), which may affect the broad generalizability of our findings. Future studies with larger, multi-center cohorts are warranted to validate and extend our observations. In summary, our single-cell multi-omics analysis elucidates the heterogeneity and metabolic adaptation of ADSCs in diabetes, a systemic chronic inflammatory condition. Our comprehensive molecular profiling provides valuable insights into the pathogenesis of diabetic ulcers. Furthermore, experimental validation revealed the regulatory mechanisms of autophagy and apoptosis in ADSCs under high glucose stress, offering potential strategies for precision stem cell therapy for diabetic wound healing.

## 4 Materials and methods

### 4.1 Single-cell data acquisition

Single-cell RNA-sequencing data (GSE229387) were obtained from the Gene Expression Omnibus (https://www.ncbi.nlm.nih.gov/geo/), comprising human ADSCs from three donors with diabetes mellitus (DM) and three without.

### 4.2 Data processing and dimensionality reduction

Raw sequencing data generated by the 10x Genomics platform were processed using Seurat (v4.3.0) to construct Seurat objects. Doublets were identified and removed using DoubletFinder (v2.0.3). Low-quality cells were filtered based on the following thresholds: 300–5000 detected genes (nFeature), 500–40,000 transcripts (nCount), <20% mitochondrial gene content, and <5% hemoglobin gene content.

Filtered cells were normalized using “NormalizeData” (53), and the top 2,000 highly variable genes were identified with “FindVariableFeatures”. Data were scaled by “ScaleData”, followed by principal component analysis (PCA) for dimensionality reduction. Batch effects were corrected using Harmony (v0.1.1) based on the top 30 principal components, which were subsequently used for downstream analyses. Uniform Manifold Approximation and Projection (UMAP) was then applied for visualization. Cell clustering was performed using “FindNeighbors” and “FindClusters” in Seurat.

### 4.3 Dimensionality reduction and clustering

Principal component analysis (PCA) was performed for dimensionality reduction (54), and the top 30 principal components were selected for downstream analyses. Uniform Manifold Approximation and Projection (UMAP) was applied for visualization (55). Cell clustering was conducted using the FindNeighbors and FindClusters functions in Seurat.

### 4.4 Subpopulation identification and differential expression analysis

Differentially expressed genes (DEGs) for each cluster were identified using FindAllMarkers, and cell subpopulations were annotated based on canonical marker genes (56, 57). To assess tissue preference across subpopulations, Ro/e values were calculated (58). Cell cycle states were quantified using the CellCycleScoring function to evaluate cell cycle distribution among subpopulations.

### 4.5 Functional enrichment analysis

Gene Ontology biological processes (GO-BP) and Kyoto Encyclopedia of Genes and Genomes (KEGG) pathway enrichment analyses (59–61) were performed using the ClusterProfiler package (v4.6.2). Gene Set Enrichment Analysis (GSEA) (62) was applied to evaluate overall expression trends of key pathways. The enrichment of stemness-related gene sets was quantified using the AUCell package (63), and gene sets were ranked with the AUCell_buildRankings function.

### 4.6 Autophagy and apoptosis analysis

Autophagy- and apoptosis- related gene sets were defined based on previously published literature (64). Pathway scores were calculated for each subpopulation to uncover potential differences in programmed cell death and autophagy regulation.

### 4.7 Metabolic pathway analysis

Metabolic activity across subpopulations was evaluated using the scMetabolism R package (65). The top 10 enriched pathways were visualized as a heatmap to characterize subpopulation-specific metabolic features.

### 4.8 Patient information

Human tissue samples were obtained from Department of Burn and Plastic Surgery, Seventh People’s Hospital Affiliated to Shanghai University of Traditional Chinese Medicine. All protocols involving human subjects were reviewed and approved by the ethics committee of the Seventh People’s Hospital Affiliated to Shanghai University of Traditional Chinese Medicine (2024-7th-HIRB-016). All procedures were carried out in accordance with guidelines set forth by Declaration of Helsinki. Written informed consents were obtained.

### 4.9 Isolation and culture of human ADSCs

Adipose tissues were washed with PBS, minced thoroughly, and digested with 0.1% collagenase (Sigma-Aldrich, USA) at 37 °C for 60 min. The digested suspension was filtered and centrifuged to isolate the stromal vascular fraction. Cells were cultured in DMEM/F12 (Gibco, USA) with 10% FBS (Gibco, USA) at 37 °C, 5% CO_2_, with medium replacement after 24 h and every 3 days thereafter.

### 4.10 Cell treatment

ADSCs were cultured with normal glucose medium (5.5mM; Gibco, USA) or high glucose medium (25 mM; Gibco, USA) for 24 h *in vitro* before subsequent analysis.

### 4.11 Immunofluorescence staining

Cells on coverslips were fixed with 4% paraformaldehyde, permeabilized with 0.1% Triton X-100, and blocked with 3% BSA. After overnight incubation with primary antibodies at 4 °C, samples were treated with Alexa Fluor 488-conjugated secondary antibody (Abcam, USA) at RT for 1h. Nuclei were stained with DAPI (Yeasen, China). Images were captured using confocal fluorescence microscopy (Leica, Germany).

### 4.12 Apoptosis assay

ADSCs were stained with FITC-Annexin V/PI (KeyGen, China) and analyzed on flow cytometer (Agilent, USA) to quantify apoptotic cells.

### 4.13 Western blot

Protein lysates were resolved by SDS-PAGE and transferred to nitrocellulose membranes. Membranes were probed with the rabbit polyclonal antibodies: anti-LC3 (1:1000; CST, USA), anti-Beclin1 (1:1000; CST, USA), anti-p62 (1:1000; CST, USA), anti-Bax (1:1000; CST, USA), anti-cleaved Caspase-3 (1:500; CST, USA), anti-c-Myb (1:500; CST, USA), anti-AURKA (1:500; CST, USA) and anti-β-actin (1:1000; CST, USA). Protein bands were visualized using Servicebio scanning system.

### 4.14 RT-qPCR

Total RNA was extracted using NcmSpin Cell/Tissue Total RNA Kit (NCM, China) and reverse transcribed with PrimeScript RT Master Mix Kit (TaKaRa, Japan). Quantitative PCR was performed using TB Green Premix Ex Taq (TaKaRa, Japan), using specific primers listed in **Supplementary Table S1**. All specific primers were obtained from Servicebio (China). Gene expression was quantified by using the 2−ΔΔCT method.

### 4.15 Co-immunoprecipitation

Cells were lysed in IP buffer and incubated with anti-c-Myb antibody (CST, USA) and protein A/G agarose beads (Santa Cruz, USA). After washing, immunocomplexes were eluted in SDS sample buffer and analyzed by immunoblotting.

### 4.16 Chromatin immunoprecipitation

ChIP were performed according to the manufacturer’s protocol (Millipore, USA). Chromatin was immunoprecipitated with anti-c-Myb or control IgG antibodies. Precipitated DNA was amplified by PCR using the following primers. Region1: forward 5’- TTT CCC TGT GCT TTC CTT AC-3’ and reverse 5’-GGT GGT GTC AGC CTC TAA TC-3’; Region2: forward 5’-AGT CTG GCA AAG AAA AGT TGA T-3’ and reverse 5’-GTG TAG GGG AAC CAA AAA TG-3’; Region3: forward 5’-GCA ACT TAG GAA ACG CAA AGT AG-3’ and reverse 5’-GAG CGG GAT ACC AAG AGA AC-3’. PCR products were resolved by agarose gel electrophoresis.

### 4.17 Luciferase reporter assay

Cells were transfected with a AURKA promoter-driven luciferase reporter construct or the control luciferase construct using Lipofectamine stem transfection reagent for 24 h. Firefly and Renilla luciferase activities were quantified using a dual luciferase kit (Servicebio, China) according to the manufacturer’s protocol.

### 4.18 shRNA and plasmid transfection

Cells were transfected with shRNAs or plasmids (Hanbio, China) using Lipofectamine 2000 (Invitrogen, USA), following the manufacturers’ instructions.

### 4.19 Statistical analysis

Data are presented as mean ± SD of three independent biological replicates and statistically analyzed utilizing a Student’s t-test for two-group comparisons or using One-way ANOVA for multi-group comparisons by using SPSS software (SPSS 16.0). In addition, R software (v4.3.0) and Python software (v3.9.0) were employed for statistical analysis and data processing. A *p*-value < 0.05 was considered statistically significant.

## Data Availability

The datasets presented in this study can be found in online repositories. The names of the repository/repositories and accession number(s) can be found in the article/**Supplementary Material**.

## Supplementary material

Supplementary Figure 1 Enrichment analysis based on DEGs. **(A)** Enrichment analysis of the 14 DM ADSCs subpopulations based on DEGs, with the top five terms displayed.

Supplementary Figure 2 GO functional annotation of C8 AURKA ADSCs. **(A)** GO functional categories of the C8 AURKA ADSCs subpopulation, including GOBP, GOCC, and GOMF.

## References

[1] Xie Y Ni X Wan X Xu N Chen L Lin C. KLF5 enhances CXCL12 transcription in adipose-derived stem cells to promote endothelial progenitor cells neovascularization and accelerate diabetic wound healing. Cell Mol Biol Lett. (2025) 30:24. doi: 10.1186/s11658-025-00702-0, PMID: 40038579 PMC11877965

[2] Tajali R Eidi A Tafti HA Pazouki A Kamarul T Sharifi AM. Transplantation of adipose derived stem cells in diabetes mellitus; limitations and achievements. J Diabetes Metab Disord. (2023) 22:1039–52. doi: 10.1007/s40200-023-01280-8, PMID: 37975135 PMC10638327

[3] Cao Y Yan J Dong Z Wang J Jiang X Cui T. Adipose-derived mesenchymal stem cells are ideal for the cell-based treatment of refractory wounds: strong potential for angiogenesis. Stem Cell Rev Rep. (2024) 20:313–28. doi: 10.1007/s12015-023-10641-y, PMID: 37874529

[4] Lu Y Gao H Wang S Xu H Chen Z Zhang Y. Integrated transcriptomic, proteomic, and metabolomic analysis unveils key roles of protein and nucleic acid interactions in diabetic ulcer pathogenesis. Front Endocrinol (Lausanne). (2025) 16:1574858. doi: 10.3389/fendo.2025.1574858, PMID: 40620796 PMC12226305

[5] Li Q Yin Y Zheng Y Chen F Jin P. Inhibition of autophagy promoted high glucose/ROS-mediated apoptosis in ADSCs. Stem Cell Res Ther. (2018) 9:289. doi: 10.1186/s13287-018-1029-4, PMID: 30359319 PMC6203262

[6] Ramsay RG Barton AL Gonda TJ. Targeting c-Myb expression in human disease. Expert Opin Ther Targets. (2003) 7:235–48. doi: 10.1517/14728222.7.2.235, PMID: 12667100

[7] Ramsay RG Ciznadija D Mantamadiotis T Anderson R Pearson R. Expression of stress response protein glucose regulated protein-78 mediated by c-Myb. Int J Biochem Cell Biol. (2005) 37:1254–68. doi: 10.1016/j.biocel.2004.12.011, PMID: 15778089

[8] Zhang Y Jin H Li L Qin FX Wen Z. cMyb regulates hematopoietic stem/progenitor cell mobilization during zebrafish hematopoiesis. Blood. (2011) 118:4093–101. doi: 10.1182/blood-2011-03-342501, PMID: 21856868

[9] Kaspar P Ilencikova K Zikova M Horvath O Cermak V Bartunek P. c-Myb inhibits myoblast fusion. PloS One. (2013) 8:e76742. doi: 10.1371/journal.pone.0076742, PMID: 24204667 PMC3804598

[10] Oralova V Matalova E Killinger M Knopfova L Smarda J Buchtova M. Osteogenic potential of the transcription factor c-MYB. Calcif Tissue Int. (2017) 100:311–22. doi: 10.1007/s00223-016-0219-2, PMID: 28012106

[11] Bai T Li M Liu Y Qiao Z Zhang X Wang Y. The promotion action of AURKA on post-ischemic angiogenesis in diabetes-related limb ischemia. Mol Med. (2023) 29:39. doi: 10.1186/s10020-023-00635-4, PMID: 36977984 PMC10053687

[12] Kim SJ Park SE Jeong JB Oh SJ Choi A Kim YH. Wharton’s jelly-derived mesenchymal stem cells with high aurora kinase A expression show improved proliferation, migration, and therapeutic potential. Stem Cells Int. (2022) 2022:4711499. doi: 10.1155/2022/4711499, PMID: 35450345 PMC9017458

[13] Ritter A Kreis NN Roth S Friemel A Jennewein L Eichbaum C. Restoration of primary cilia in obese adipose-derived mesenchymal stem cells by inhibiting Aurora A or extracellular signal-regulated kinase. Stem Cell Res Ther. (2019) 10:255. doi: 10.1186/s13287-019-1373-z, PMID: 31412932 PMC6694567

[14] Liao N Shi Y Zhang C Zheng Y Wang Y Zhao B. Antioxidants inhibit cell senescence and preserve stemness of adipose tissue-derived stem cells by reducing ROS generation during long-term *in vitro* expansion. Stem Cell Res Ther. (2019) 10:306. doi: 10.1186/s13287-019-1404-9, PMID: 31623678 PMC6798439

[15] Tu C Lu H Zhou T Zhang W Deng L Cao W. Promoting the healing of infected diabetic wound by an anti-bacterial and nano-enzyme-containing hydrogel with inflammation-suppressing, ROS-scavenging, oxygen and nitric oxide-generating properties. Biomaterials. (2022) 286:121597. doi: 10.1016/j.biomaterials.2022.121597, PMID: 35688112

[16] Worsley AL Lui DH Ntow-Boahene W Song W Good L Tsui J. The importance of inflammation control for the treatment of chronic diabetic wounds. Int Wound J. (2023) 20:2346–59. doi: 10.1111/iwj.14048, PMID: 36564054 PMC10333011

[17] Teh HX Phang SJ Looi ML Kuppusamy UR Arumugam B. Molecular pathways of NF-kB and NLRP3 inflammasome as potential targets in the treatment of inflammation in diabetic wounds: A review. Life Sci. (2023) 334:122228. doi: 10.1016/j.lfs.2023.122228, PMID: 37922981

[18] Lin S Zhan Y Wang R Pei J. Decoding neuroinflammation in Alzheimer’s disease: a multi-omics and AI-driven perspective for precision medicine. Front Immunol. (2025) 16:1616899. doi: 10.3389/fimmu.2025.1616899, PMID: 40607399 PMC12213348

[19] Fang S Zhang H Liu W Li S Chen Z Min J. Analysis and validation of mitophagy-related genes in diabetic foot ulcers. J Inflammation Res. (2025) 18:4367–79. doi: 10.2147/JIR.S504001, PMID: 40162084 PMC11954483

[20] Xin X Zhou H Huang S Zhang W Xu J Wang W. Identification of biomarkers and potential drug targets in DFU based on fundamental experiments and multi-omics joint analysis. Front Pharmacol. (2025) 16:1561179. doi: 10.3389/fphar.2025.1561179, PMID: 40487403 PMC12141248

[21] Wang Z Wei D Li S Tang Q Lu G Gu S. Healing mechanism of diabetic foot ulcers using single-cell RNA-sequencing. Ann Transl Med. (2023) 11:210. doi: 10.21037/atm-23-240, PMID: 37007553 PMC10061471

[22] Theocharidis G Thomas BE Sarkar D Mumme HL Pilcher WJR Dwivedi B. Single cell transcriptomic landscape of diabetic foot ulcers. Nat Commun. (2022) 13:181. doi: 10.1038/s41467-021-27801-8, PMID: 35013299 PMC8748704

[23] Dong Y Wang M Wang Q Cao X Chen P Gong Z. Single-cell RNA-seq in diabetic foot ulcer wound healing. Wound Repair Regener. (2024) 32:880–9. doi: 10.1111/wrr.13218, PMID: 39264020 PMC11584366

[24] Qiao J Zhou H Wang J Wang J Zhong L Chen J. Analysis of ferroptosis-related key genes and regulatory networks in diabetic foot ulcers. Gene. (2025) 950:149375. doi: 10.1016/j.gene.2025.149375, PMID: 40024299

[25] Meng F Sun Q Zhou D Li Q Han J Liu D. Inhibition of Aurora-A improves insulin resistance by ameliorating islet inflammation and controlling interleukin-6 in a diabetic mouse model. Adipocyte. (2020) 9:609–19. doi: 10.1080/21623945.2020.1829851, PMID: 33043822 PMC7553512

[26] Moujalled D Strasser A Liddell JR. Molecular mechanisms of cell death in neurological diseases. Cell Death Differ. (2021) 28:2029–44. doi: 10.1038/s41418-021-00814-y, PMID: 34099897 PMC8257776

[27] Yin Y Chen F Li J Yang J Li Q Jin P. AURKA enhances autophagy of adipose derived stem cells to promote diabetic wound repair via targeting FOXO3a. J Invest Dermatol. (2020) 140:1639–49 e4. doi: 10.1016/j.jid.2019.12.032, PMID: 32004564

[28] Asteriti IA De Mattia F Guarguaglini G. Cross-talk between AURKA and plk1 in mitotic entry and spindle assembly. Front Oncol. (2015) 5:283. doi: 10.3389/fonc.2015.00283, PMID: 26779436 PMC4688340

[29] Ren H Zhao F Zhang Q Huang X Wang Z. Autophagy and skin wound healing. Burns Trauma. (2022) 10:tkac003. doi: 10.1093/burnst/tkac003, PMID: 35187180 PMC8847901

[30] Li L Zhang J Zhang Q Zhang D Xiang F Jia J. High glucose suppresses keratinocyte migration through the inhibition of p38 MAPK/autophagy pathway. Front Physiol. (2019) 10:24. doi: 10.3389/fphys.2019.00024, PMID: 30745880 PMC6360165

[31] Zhou KL Zhou YF Wu K Tian NF Wu YS Wang YL. Stimulation of autophagy promotes functional recovery in diabetic rats with spinal cord injury. Sci Rep. (2015) 5:17130. doi: 10.1038/srep17130, PMID: 26597839 PMC4657088

[32] Song FC Yuan JQ Zhu MD Li Q Liu SH Zhang L. High glucose represses the proliferation of tendon fibroblasts by inhibiting autophagy activation in tendon injury. Biosci Rep. (2022) 42(3):BSR20210640. doi: 10.1042/BSR20210640, PMID: 35293974 PMC8935382

[33] Huang H Liang L Sun D Li J Wang W Zha L. Rab37 promotes endothelial differentiation and accelerates ADSC-mediated diabetic wound healing through regulating secretion of hsp90alpha and TIMP1. Stem Cell Rev Rep. (2023) 19:1019–33. doi: 10.1007/s12015-022-10491-0, PMID: 36627432

[34] Ren H Su P Zhao F Zhang Q Huang X He C. Adipose mesenchymal stem cell-derived exosomes promote skin wound healing in diabetic mice by regulating epidermal autophagy. Burns Trauma. (2024) 12:tkae001. doi: 10.1093/burnst/tkae001, PMID: 38434722 PMC10905655

[35] Li K Deng Y Deng G Chen P Wang Y Wu H. High cholesterol induces apoptosis and autophagy through the ROS-activated AKT/FOXO1 pathway in tendon-derived stem cells. Stem Cell Res Ther. (2020) 11:131. doi: 10.1186/s13287-020-01643-5, PMID: 32197645 PMC7082977

[36] He R Wang Z Cui M Liu S Wu W Chen M. HIF1A Alleviates compression-induced apoptosis of nucleus pulposus derived stem cells via upregulating autophagy. Autophagy. (2021) 17:3338–60. doi: 10.1080/15548627.2021.1872227, PMID: 33455530 PMC8632345

[37] Ouyang L Qiu D Fu X Wu A Yang P Yang Z. Overexpressing HPGDS in adipose-derived mesenchymal stem cells reduces inflammatory state and improves wound healing in type 2 diabetic mice. Stem Cell Res Ther. (2022) 13:395. doi: 10.1186/s13287-022-03082-w, PMID: 35922870 PMC9351105

[38] Hu N Cai Z Jiang X Wang C Tang T Xu T. Hypoxia-pretreated ADSC-derived exosome-embedded hydrogels promote angiogenesis and accelerate diabetic wound healing. Acta Biomater. (2023) 157:175–86. doi: 10.1016/j.actbio.2022.11.057, PMID: 36503078

[39] Li CW Young TH Wang MH Pei MY Hsieh TY Hsu CL. Low-glucose culture environment can enhance the wound healing capability of diabetic adipose-derived stem cells. Stem Cell Res Ther. (2023) 14:236. doi: 10.1186/s13287-023-03478-2, PMID: 37667384 PMC10478288

[40] Song Y You Y Xu X Lu J Huang X Zhang J. Adipose-derived mesenchymal stem cell-derived exosomes biopotentiated extracellular matrix hydrogels accelerate diabetic wound healing and skin regeneration. Adv Sci (Weinh). (2023) 10:e2304023. doi: 10.1002/advs.202304023, PMID: 37712174 PMC10602544

[41] Lin Z Li LY Chen L Jin C Li Y Yang L. Lonicerin promotes wound healing in diabetic rats by enhancing blood vessel regeneration through Sirt1-mediated autophagy. Acta Pharmacol Sin. (2024) 45:815–30. doi: 10.1038/s41401-023-01193-5, PMID: 38066346 PMC10943091

[42] Liang D Lin WJ Ren M Qiu J Yang C Wang X. m(6)A reader YTHDC1 modulates autophagy by targeting SQSTM1 in diabetic skin. Autophagy. (2022) 18:1318–37. doi: 10.1080/15548627.2021.1974175, PMID: 34657574 PMC9225222

[43] Kopecki Z Luchetti MM Adams DH Strudwick X Mantamadiotis T Stoppacciaro A. Collagen loss and impaired wound healing is associated with c-Myb deficiency. J Pathol. (2007) 211:351–61. doi: 10.1002/path.2113, PMID: 17152050

[44] Lee YH Kim HS Kim JS Yu MK Cho SD Jeon JG. C-myb regulates autophagy for pulp vitality in glucose oxidative stress. J Dent Res. (2016) 95:430–8. doi: 10.1177/0022034515622139, PMID: 26661713

[45] Zhou P Feng H Qin W Li Q. KRT17 from skin cells with high glucose stimulation promotes keratinocytes proliferation and migration. Front Endocrinol (Lausanne). (2023) 14:1237048. doi: 10.3389/fendo.2023.1237048, PMID: 37929023 PMC10622786

[46] Chen Y Zhao X Wu H. Metabolic stress and cardiovascular disease in diabetes mellitus: the role of protein O-glcNAc modification. Arterioscler Thromb Vasc Biol. (2019) 39:1911–24. doi: 10.1161/ATVBAHA.119.312192, PMID: 31462094 PMC6761006

[47] Ighodaro OM. Molecular pathways associated with oxidative stress in diabetes mellitus. BioMed Pharmacother. (2018) 108:656–62. doi: 10.1016/j.biopha.2018.09.058, PMID: 30245465

[48] Semenza GL. HIF-1 mediates metabolic responses to intratumoral hypoxia and oncogenic mutations. J Clin Invest. (2013) 123:3664–71. doi: 10.1172/JCI67230, PMID: 23999440 PMC3754249

[49] Erukainure OL Reddy R Islam MS. Raffia palm (Raphia hookeri) wine extenuates redox imbalance and modulates activities of glycolytic and cholinergic enzymes in hyperglycemia-induced testicular injury in type 2 diabetic rats. J Food Biochem. (2019) 43:e12764. doi: 10.1111/jfbc.12764, PMID: 31353550

[50] Rato L Alves MG Dias TR Cavaco JE Oliveira PF. Testicular metabolic reprogramming in neonatal streptozotocin-induced type 2 diabetic rats impairs glycolytic flux and promotes glycogen synthesis. J Diabetes Res. (2015) 2015:973142. doi: 10.1155/2015/973142, PMID: 26064993 PMC4443934

[51] Fleming TH Theilen TM Masania J Wunderle M Karimi J Vittas S. Aging-dependent reduction in glyoxalase 1 delays wound healing. Gerontology. (2013) 59:427–37. doi: 10.1159/000351628, PMID: 23797271

[52] Miyazawa N Abe M Souma T Tanemoto M Abe T Nakayama M. Methylglyoxal augments intracellular oxidative stress in human aortic endothelial cells. Free Radic Res. (2010) 44:101–7. doi: 10.3109/10715760903321788, PMID: 19886746

[53] Lin Z Zhou Y Liu Z Nie W Cao H Li S. Deciphering the tumor immune microenvironment: single-cell and spatial transcriptomic insights into cervical cancer fibroblasts. J Exp Clin Cancer Res. (2025) 44:194. doi: 10.1186/s13046-025-03432-5, PMID: 40616092 PMC12228347

[54] Zhao Z Cai H Nie W Wang X Zhao Z Zhao F. Ectopic expression of GDF15 in cancer-associated fibroblasts enhances melanoma immunosuppression via the GFRAL/RET cascade. J Immunother Cancer. (2025) 13(6):e011036. doi: 10.1136/jitc-2024-011036, PMID: 40555562 PMC12198796

[55] Nie W Zhao Z Xiahou Z Zhang J Liu Y Wang Y. Single-cell RNA sequencing reveals the potential role of Postn(+) fibroblasts in promoting the progression of myocardial fibrosis after myocardial infarction. Sci Rep. (2025) 15:22390. doi: 10.1038/s41598-025-04990-6, PMID: 40595870 PMC12217889

[56] Ding Y Xiao L Zhou X Zhao J Ke J Cai H. Molecular insights into glioblastoma progression: role of CHCHD2P9 in tumor heterogeneity and prognosis. Front Immunol. (2025) 16:1581850. doi: 10.3389/fimmu.2025.1581850, PMID: 40630954 PMC12234496

[57] Zhou H Jing S Liu Y Wang X Duan X Xiong W. Identifying the key genes of Epstein-Barr virus-regulated tumour immune microenvironment of gastric carcinomas. Cell Prolif. (2023) 56:e13373. doi: 10.1111/cpr.13373, PMID: 36519208 PMC9977676

[58] Zheng L Qin S Si W Wang A Xing B Gao R. Pan-cancer single-cell landscape of tumor-infiltrating T cells. Science. (2021) 374:abe6474. doi: 10.1126/science.abe6474, PMID: 34914499

[59] Sun Y Nie W Xiahou Z Wang X Liu W Liu Z. Integrative single-cell and spatial transcriptomics uncover ELK4-mediated mechanisms in NDUFAB1+ tumor cells driving gastric cancer progression, metabolic reprogramming, and immune evasion. Front Immunol. (2025) 16:1591123. doi: 10.3389/fimmu.2025.1591123, PMID: 40688093 PMC12271198

[60] Li XL Lin ZH Chen SR Ni S Lin GY Wang W. Tiaogeng decoction improves mild cognitive impairment in menopausal APP/PS1 mice through the ERs/NF-kappa b/AQP1 signaling pathway. Phytomedicine. (2025) 138:156391. doi: 10.1016/j.phymed.2025.156391, PMID: 39848022

[61] Lin Z Fan W Yu X Liu J Liu P. Research into the mechanism of intervention of SanQi in endometriosis based on network pharmacology and molecular docking technology. Med (Baltimore). (2022) 101:e30021. doi: 10.1097/MD.0000000000030021, PMID: 36123943 PMC9478308

[62] Zhao Z Dong Y Zhao Z Xiahou Z Sun C. Single-cell atlas of endothelial cells in atherosclerosis: identifying C1 CXCL12+ ECs as key proliferative drivers for immunological precision therapeutics in atherosclerosis. Front Immunol. (2025) 16:1569988. doi: 10.3389/fimmu.2025.1569988, PMID: 40421026 PMC12104226

[63] Zhao Z Zhao Z Lin Z Fan L Xiahou Z Dong Y. Decoding multiple myeloma: single-cell insights into tumor heterogeneity, immune dynamics, and disease progression. Front Immunol. (2025) 16:1584350. doi: 10.3389/fimmu.2025.1584350, PMID: 40406148 PMC12095158

[64] Zou Y Xie J Zheng S Liu W Tang Y Tian W. Leveraging diverse cell-death patterns to predict the prognosis and drug sensitivity of triple-negative breast cancer patients after surgery. Int J Surg. (2022) 107:106936. doi: 10.1016/j.ijsu.2022.106936, PMID: 36341760

[65] Liu P Xing N Xiahou Z Yan J Lin Z Zhang J. Unraveling the intricacies of glioblastoma progression and recurrence: insights into the role of NFYB and oxidative phosphorylation at the single-cell level. Front Immunol. (2024) 15:1368685. doi: 10.3389/fimmu.2024.1368685, PMID: 38510250 PMC10950940

