# Supplementary figures and images for: Single-cell multi-omics dissection of c-Myb/AURKA-mediated autophagy and metabolic reprogramming in diabetic adipose-derived stem cells
Source: Front Immunol. 2025 Sep 25;16:1665909. doi: 10.3389/fimmu.2025.1665909 (PMC12507903; doi:10.3389/fimmu.2025.1665909)

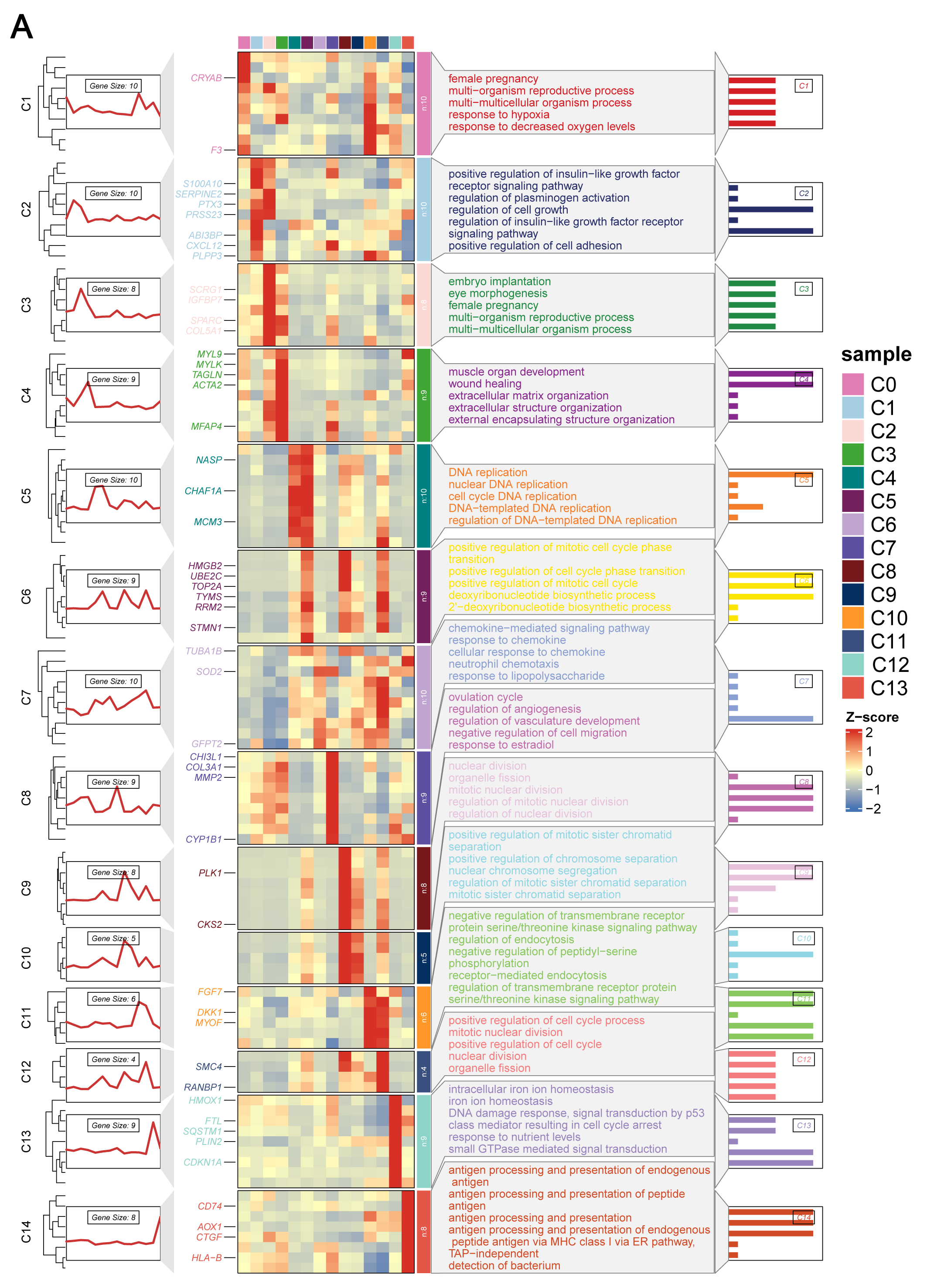

Supplement: Supplementary Figure 1 — Enrichment analysis based on DEGs. (A) Enrichment analysis of the 14 DM ADSCs subpopulations based on DEGs, with the top five terms displayed. [file Image1.tif]

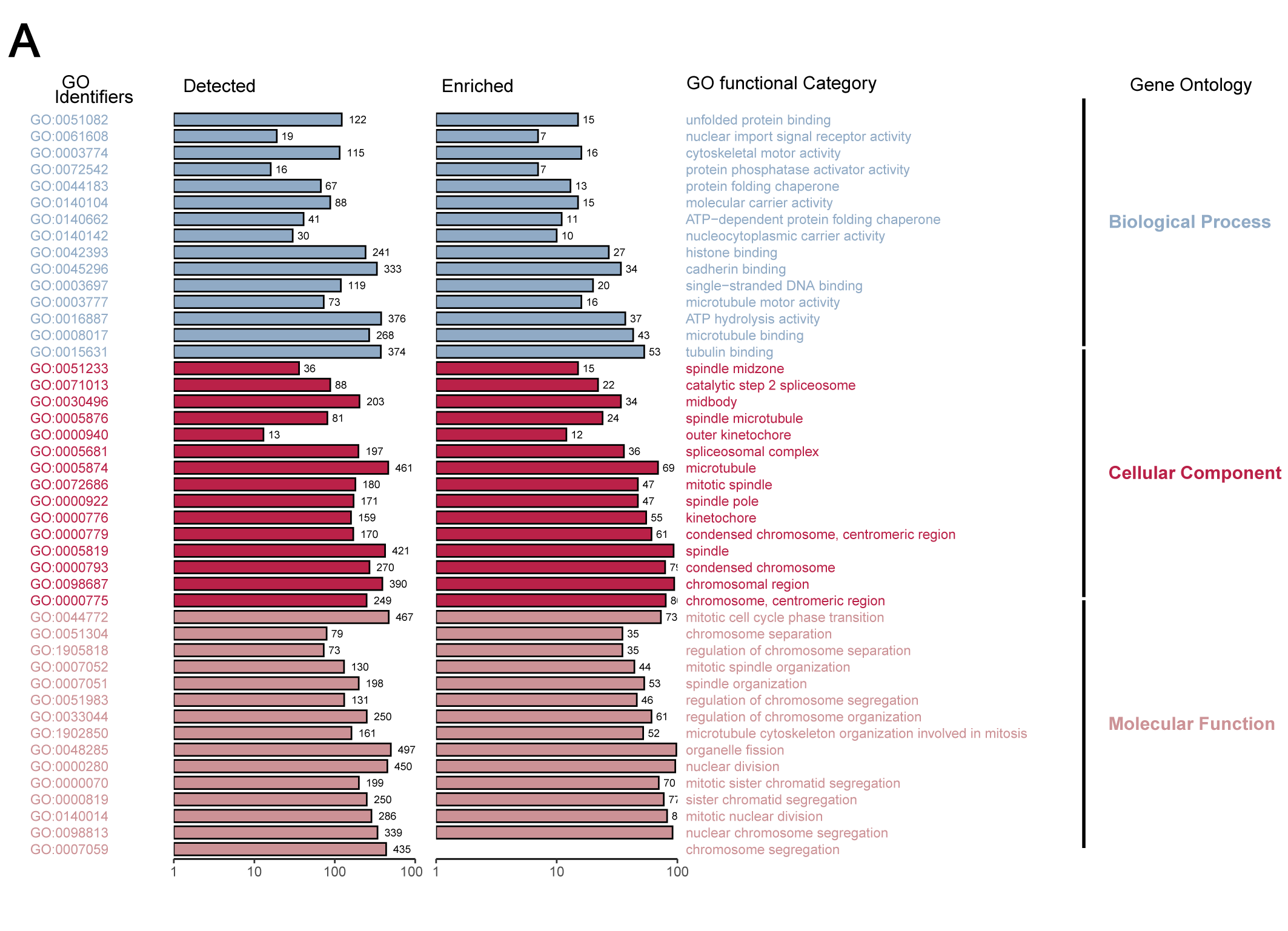

Supplement: Supplementary Figure 2 — GO functional annotation of C8 AURKA ADSCs. (A) GO functional categories of the C8 AURKA ADSCs subpopulation, including GOBP, GOCC, and GOMF. [file Image2.tif]
