# Supplementary material for: Single-cell multi-omics dissection of c-Myb/AURKA-mediated autophagy and metabolic reprogramming in diabetic adipose-derived stem cells
Source: Front Immunol. 2025 Sep 25;16:1665909. doi: 10.3389/fimmu.2025.1665909 (PMC12507903; doi:10.3389/fimmu.2025.1665909)
Supplement: Supplementary file 3 [file Table1.docx]

**Table S1: Quantitative PCR amplification primers**

| **Gene name** | **Forward primer** | **Reversed primer** |
| --- | --- | --- |
| β-ACTIN | CACCCAGCACAATGAAGATCAAGAT | CCAGTTTTTAAATCCTGAGTCAAGC |
| LC3B | AACGATACAAGGGTGAGAAGCA | CACTGACAATTTCATCCCGAAC |
| BECLIN1 | GGCACAATCAATAACTTCAGGC | CCGTAAGGAACAAGTCGGTATCTC |
| ATG7 | TAGTAGTGCCTTGGATGTTGGG | CAGCCCAGCAGAGTCACCATT |
| ATG5 | GGCCATCAATCGGAAACTCA | CGGGTAGCTCAGATGTTCACTC |
| C-MYB | AATGGACCACCCTTACTGAAGAAAA | GGTCTGCGTGAACAGTTGGGT |
| AURKA | AGTCCCACCTTCGGCATCCTA | GAATGACAGTAAGACAGGGCATTTG |
